# Muscle fatigue assessment during robot-mediated movements

**DOI:** 10.1186/s12984-018-0463-y

**Published:** 2018-12-17

**Authors:** Maddalena Mugnosso, Francesca Marini, Michael Holmes, Pietro Morasso, Jacopo Zenzeri

**Affiliations:** 1 Motor Learning, Assistive and Rehabilitation Robotics Lab, Robotics, Brain and Cognitive Sciences unit, Istituto Italiano di Tecnologia, Genoa, Italy; 2 Department of Informatics, Bioengineering, Robotics and Systems Engineering (DIBRIS),University of Genoa, Genoa, Italy; 3 Department of Kinesiology, Brock University, St. Catharines, ON Canada

**Keywords:** Fatigue, sEMG, Robot-aided assessment, Neuromuscular disorders, Robotic rehabilitation, Upper limb

## Abstract

**Background:**

Several neuromuscular disorders present muscle fatigue as a typical symptom. Therefore, a reliable method of fatigue assessment may be crucial for understanding how specific disease features evolve over time and for developing effective rehabilitation strategies. Unfortunately, despite its importance, a standardized, reliable and objective method for fatigue measurement is lacking in clinical practice and this work investigates a practical solution.

**Methods:**

40 healthy young adults performed a haptic reaching task, while holding a robotic manipulandum. Subjects were required to perform wrist flexion and extension movements in a resistive visco-elastic force field, as many times as possible, until the measured muscles (mainly *flexor* and *extensor carpi radialis*) exhibited signs of fatigue. In order to analyze the behavior and the characteristics of the two muscles, subjects were divided into two groups: in the first group, the resistive force was applied by the robot only during flexion movements, whereas, in the second group, the force was applied only during extension movements. Surface electromyographic signals (sEMG) of both *flexor* and *extensor carpi radialis* were acquired. A novel indicator to define the *Onset of Fatigue (OF)* was proposed and evaluated from the *Mean Frequency* of the sEMG signal. Furthermore, as measure of the subjects’ effort throughout the task, the energy consumption was estimated.

**Results:**

From the beginning to the end of the task, as expected, all the subjects showed a decrement in *Mean Frequency* of the muscle involved in movements resisting the force. For the *OF* indicator, subjects were consistent in terms of timing of fatigue; moreover, *extensor* and *flexor* muscles presented similar *OF* times. The metabolic analysis showed a very low level of energy consumption and, from the behavioral point of view, the test was well tolerated by the subjects.

**Conclusion:**

The robot-aided assessment test proposed in this study, proved to be an easy to administer, fast and reliable method for objectively measuring muscular fatigue in a healthy population. This work developed a framework for an evaluation that can be deployed in a clinical practice with patients presenting neuromuscular disorders. Considering the low metabolic demand, the requested effort would likely be well tolerated by clinical populations.

## Background

Muscle fatigue has been defined as “the failure to maintain a required or expected force” [1] and it is a complex phenomenon experienced in everyday life that has reached great interest in the areas of sports, medicine and ergonomics [2]. Muscle fatigue can affect task performance, posture-movement coordination [3], position sense [4] and it can be a highly debilitating symptom in several pathologies [5]. For many patients with neuromuscular impairments, taking into account muscle fatigue is of crucial importance in the design of correct rehabilitation protocols [6] and fatigue assessment can provide crucial information about skeletal muscle function. Specifically, several neuromuscular diseases (e.g. Duchenne, Becker Muscular Dystrophies, and spinal muscular atrophy) present muscle fatigue as a typical symptom [7], and fatigue itself accounts for a significant portion of the disease burden. A systematic approach to assess muscle fatigue might provide important cues on the disability itself, on its progression and on the efficacy of adopted therapies. In particular, therapeutic strategies are now under deep investigation and a lot of effort has been devoted to accelerate the development of drugs targeting these disorders [8]. Therefore, the need for an objective tool to measure muscle fatigue is impelling and of great relevance.

Currently, in clinical practice muscle fatigue is evaluated by means of qualitative rating scales like the 6-min walk test (6MWT) [9] or through subjective questionnaires administered to the patient (e.g. the Multidimensional Fatigue Inventory (MFI), the Fatigue Severity Scale (FSS), and the Visual Analog Scale (VAS)) [10]. During the 6MWT patients have to walk, as fast as possible, along a 25 meters linear course and repeat it as often as they can for 6 min: ‘fatigue’ is then defined as the difference between the distance covered in the sixth minute compared to the first. Obviously, such a measure is only applicable to ambulant patients and this is a strong limitation to clinical investigation because a patient may lose ambulatory ability during a clinical trial, resulting in lost ability to perform the primary clinical endpoint [11]. It should also be considered that neuromuscular patients, e.g. subjects with Duchenne Muscular Dystrophy, generally lose ambulation before 15 years of age [12], excluding a large part of the population from the measurement of fatigue through the 6MWT. Since neuromuscular patients often experience a progressive weakness also in the upper limb, reporting of muscle fatigue in this region is common. A fatigue assessment for upper limb muscles could be used to monitor patients across different stages of the disease. As for the questionnaires, the MFI is a 20 items scale designed to evaluate five dimensions of fatigue (general fatigue, physical fatigue, reduced motivation, reduced activity, and mental fatigue) [13]. Similarly, the FSS questionnaire contains nine statements that rate the severity of fatigue symptoms and the patient has to agree or disagree with them [14]. The VAS is even more general: the patient has to indicate on a 10 cm line ranging from “no fatigue” to “severe fatigue” the point that best describes his/her level of fatigue [15]. Despite the ease to administer, such subjective assessments of fatigue may not correlate with the actual severity or characteristics of fatigue, and may provide just qualitative information with low resolution, reliability and objectivity. Considering various levels of efficacy among the methods currently used in clinical practice, research should focus on the development of an assessment tool for muscle fatigue, that is easy and fast to administer, even to patients with a high level of impairment. Such a tool, should provide clear results, be easy to read and understand by a clinician, be reliable and objectively correlated with the physiology of the phenomenon.

In general, muscle fatigue can manifest from either central and/or peripheral mechanisms. Under controlled conditions, surface electromyography (sEMG) is a non-invasive and widely used technique to evaluate muscle fatigue [16]. Certain characteristics of the sEMG signal can be indicators of muscle fatigue. For example during sub-maximal tasks, muscle fatigue will present with decreases in muscle fiber conduction velocity and frequency and increases in amplitude of the sEMG signal [16]. The trend and rate of change will depend on the intensity of the task: generally, sEMG amplitude has been observed to increase during sub-maximal efforts and decrease during maximal efforts; further it has been reported that there is a significantly greater decline in the frequency content of the signal during maximal efforts compared to sub-maximal [17]. Accordingly, spectral (i.e. mean frequency) and amplitude parameters (i.e. Root Mean Square (RMS)) of the signals, can be used to measure muscle fatigue as extensively discussed in many widely acknowledged studies [16, 18, 19], however, context of contraction type and intensity must be specified for proper interpretation. A significant problem with the majority of existing protocols is that they rely on quantifying maximal voluntary force loss, maximum voluntary muscle contraction (MVC) [18, 20, 21] or high fatiguing dynamic tasks [19, 22] that cannot be reliably performed in clinical practice, especially in the case of pediatric subjects. Actually, previous works pointed out that not only the capacity to maintain MVC can be limited by a lack of cooperation [23, 24], but also, that sustaining a maximal force in isometric conditions longer than 30 s reduces subject’s motivation leading to unreliable results [25]. Besides, neuromuscular patients might have a high level of impairment and low residual muscular function thus making even more difficult, as well as dangerous for their muscles, sustaining high levels of effort or the execution of a true MVC. In order to overcome this issue, maximal muscle contractions can be elicited by magnetic [10] or electrical stimulation [26]. Although such procedures allow to bypass the problem mentioned above, these involve involuntary muscle activation and not physiological recruitment of motor units [24]; moreover, they can be uncomfortable for patients and can require advanced training, which makes them difficult to be included in clinical fatigue assessment protocols. As for the above mentioned problem with children motivation, work by Naughton et al. [27] showed that the test-retest coefficient of variation of fatigue index during a Wing-Gate test, significantly decreased when using a computerized feedback game linked to pedal cadence, suggesting that game-based procedures may ensure more consistent results in children assessment.

In recent years, the assessment of sensorimotor function has been deepened thanks to the introduction of innovative protocols administered through robotic devices [28–31]. These methods have the ambition to add meaningful information to the existing clinical scales and can be exploited as a basis for the implementation of a muscle fatigue assessment protocol. In order to fill the gap between the need of a quantitative clinical measurement protocol of muscle fatigue and the lack of an objective method which does not demand a high level of muscle activity, we propose a new method based on a robotic test, which is fast and easy to administer. Further, we decided to address the analysis of muscle fatigue on the upper limb as to provide a test suitable to assess patients from the beginning to the late stages of the disease, regardless of walking ability. Moreover, we focused on an isolated wrist flexion/extension tasks to assess wrist muscle fatigue. This ensured repeatability of the tests and prevented the adoption of compensatory movements or poor postures that may occur in multi-segmental tasks, involving the shoulder-elbow complex. In the present work, we tested the method on healthy subjects with the specific goal to evaluate when during the test the first meaningful symptoms of fatigue appaered and not how much subjects are fatigued at the end of the test. The most relevant and novel features of the proposed test include the ability to perform the test regardless of the subjects’ capability and strength, the objectivity and repeatability of the data it provides, and the simplicity and minimal time required to administer.

## Methods

### Partecipants

Forty healthy subjects with no history of motor disorders were enrolled in the study. All participants were right-handed according to the Edinburgh Handedness Inventory [32]. The study was approved by the Ethics Committee of the regional health authority, Azienda Sanitaria Locale Genovese (ASL) N.3 (Protocol number 311REG2014 approved on 09/12/2015), and all participants signed a written informed consent. Experiments were carried out at the Motor Learning, Assistive and Rehabilitation Robotics Lab of the Istituto Italiano di Tecnologia (Genoa, Italy). Participants were randomly divided into two equal groups: *Flexion Group* (*FG*) (5 male and 15 females, mean age 31.4 ± 6.3 years); and *Extension Group* (*EG*) (8 male and 12 females, mean age 25.5 ± 3.9 years). Moreover, the maximum grip force of each subject was evaluated using a hand held hydraulic dynamometer (Baseline ^*Ⓡ*^ 7-Piece Hand Evaluation Kit, Fabrication Enterprises Inc). Subject demographics are summarized in Table 1.

**Table 1:** Subject details for the *Flexion Group* (*FG*) and the *Extension Group* (*EG*): Sex, Age and hand grip force

	*FG*			*EG*		
	Sex	Age	Force [Kg]	Sex	Age	Force [Kg]
S1	F	26	21	F	21	32
S2	F	22	24	M	30	38
S3	F	26	26	M	25	40
S4	F	34	30	M	23	40
S5	F	22	25	F	33	22
S6	F	26	32	M	22	40
S7	M	35	38	F	31	36
S8	F	27	31	F	25	40
S9	M	35	40	F	26	20
S10	F	40	24	F	30	35
S11	F	37	15	F	25	35
S12	M	40	39	F	24	36
S13	F	34	25	F	30	26
S14	F	30	35	M	19	36
S15	M	43	42	M	19	34
S16	F	33	34	M	22	43
S17	F	24	36	F	26	34
S18	M	32	39	M	28	43
S19	F	37	22	F	26	27
S20	F	25	35	F	26	30

### Task and procedure

**Fig. 1:**
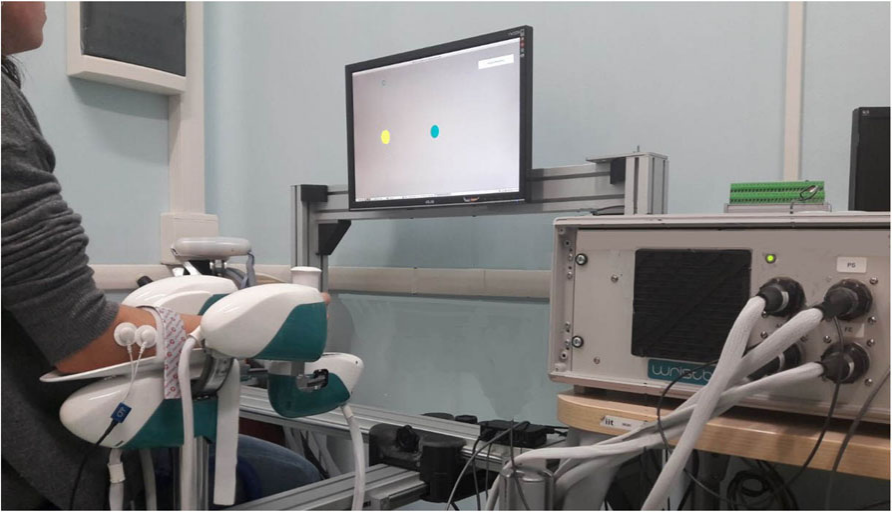
Experimental setup. Participant sitting on a chair with the forearm secured to the WRISTBOT while performing the wrist rotation reaching task. The visual targets of the reaching task are shown on a dedicated screen

**Fig. 2:**
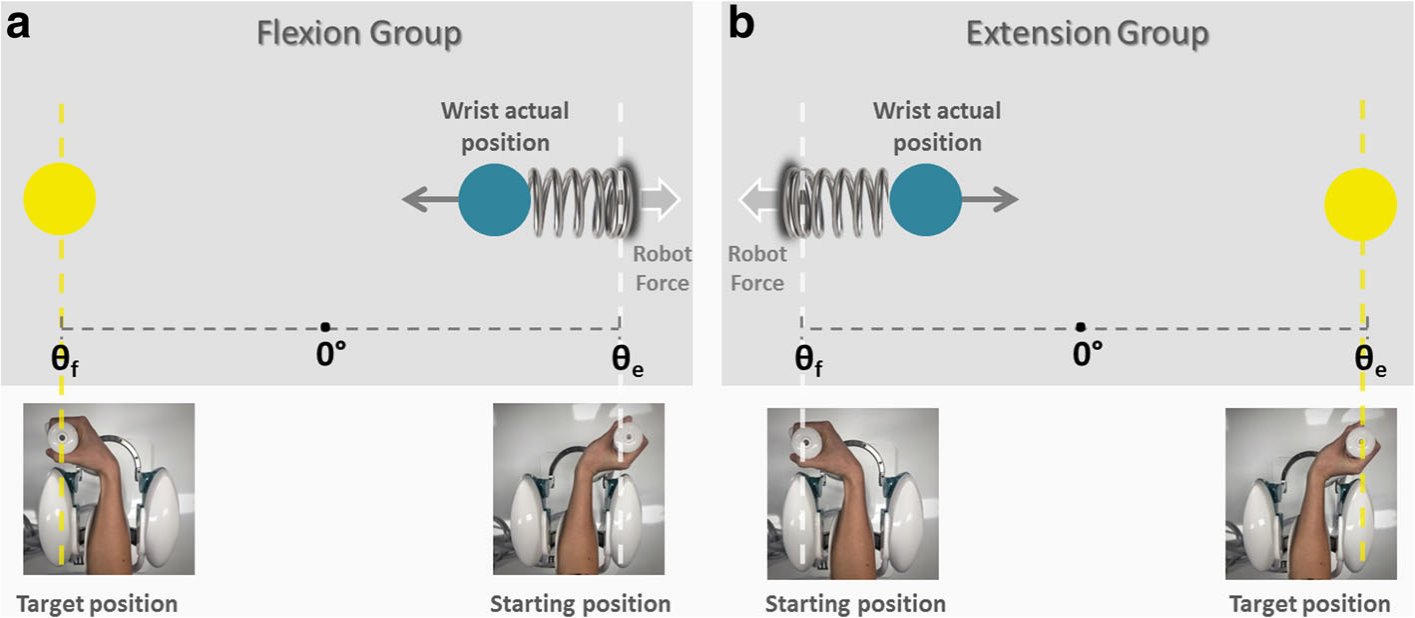
Fatigue test. Scheme of the task for the flexion group (FG) during a flexion movement (Panel **a**) and the extension group (EG) during an extension movement (Panel **b**). The blue circle represents the end-effector of the subject, the yellow circle is the target to reach

The experimental design involved a motor task where subjects were seated in front of a three degrees of freedom wrist robotic manipulandum, called WRISTBOT and developed at Istituto Italiano di Tecnologia [33, 34], holding the handle with their rigth hand (Fig. 1). Subjects’ forearm was strapped to the robot support in order to avoid forearm movements and to have a correct alignment between the axes of the mechanical structure and wrist. The device is a fully back-drivable manipulandum that has been specifically designed for human motor control studies and for sensorimotor rehabilitation. The robot allows movements along the three wrist articulations with a range of motion (RoM) similar to a typical human subject: ±62° flexion/extension (FE), 45°/- 40° radial/ulnar deviation (RUD), and ±60° pronation/supination (PS). It is powered by four brushless motors chosen in such a way to provide an accurate haptic rendering and compensate for the weight and inertia of the device. These motors can provide a maximum torque of 1.57 Nm on FE, 3.81 Nm on RUD, and 2.87 Nm on PS. Angular rotations on the three axes were acquired by means of high-resolution incremental encoders with a maximum error of ±0.17°. A visual virtual reality environment was integrated in the system in order to provide visual feedback to the users while they complete the tasks. The experimental setup was complemented by a 6-axis force/torque sensor (Optoforce HEX-58-RE-400N) mounted on the handle in order to evaluate the efficiency of the robot in terms of provided torque. The task consisted of a series of continuous target reaching movements interacting with a visco-elastic force generated by the WRISTBOT (Fig. 2). Grasping the handle of the robot, subjects were requested to perform flexion and extension movements with their wrist in order to reach Flexion/Extension targets, presented alternately at an angular distance of *θ*_*e*_=48° or *θ*_*f*_=−48° with respect to the neutral wrist position. The visco-elastic force experienced by *FG* subjects opposed flexion movements and facilitated extension movements and it was implemented as a virtual spring whose equilibrium angle was *θ*=*θ*_*e*_=48°. In the case of the *EG* subjects the visco-elastic force opposed extension movements and facilitated flexion movements, by means of a virtual spring whose equilibrium angle was *θ*=*θ*_*f*_=−48°. In both cases, a small viscous force was added, in order to provide a minimal degree of damping of the inertia of the hand:

1 $$ \left\{\begin{array}{l} F_{{FG}}=-k(\theta - \theta_{e})-b\dot{\theta} \\ F_{{EG}}=+k(\theta - \theta_{f})+b\dot{\theta} \end{array}\right.  $$

where *k* is the stiffness coefficient of the force-field and *b* the corresponding viscous coefficient. The stiffness parameter *k* was set, prior to the experiment, taking into account the significant grip force difference between male and female subjects (see Table 1). Mean female grip force was 30% less than that of males, which is in agreement with what is reported in the literature [35, 36]. More specifically, the following values of the visco-elastic parameters were chosen: *k* = 22.2 N/rad for female subjects, *k* = 27.7 N/rad for males, and *b* = 1.77 Ns/rad for all subjects. In order to avoid rest or recovery between each movements and to limit the variability between movement duration, targets had to be reached within a fixed time. In particular, visual and auditory feedback was provided: the target color changed and a sound was produced if the subject did not reach the target within a duration of 1.5 s. Besides, the minimum time was not imposed, we allowed subjects to modulate their pace as to investigate if any change in kinematic parameters occurred with muscle fatigue. Subjects were instructed to perform the target reaching task until they could no longer do the task. At this time, we considered the level of fatigue experienced by the subject to be maximal (at least by our definition) and this corresponds to the maximum score on the VAS scale [15]. During the execution of the task, the experimenter encouraged participants to perform as many repetitions as possible to assure the maximum level of acceptable fatigue had been reached. Therefore, for each subject the number of repeated ‘task-movements’ *N* could be different and the sequence duration for each subject was normalized, 0–100% (rather than 1-*N* movements) to compare subjects. During task execution, the RUD and PS degrees of freedom were haptically blocked in order to constrain movements only to the flexion/extension. Throughout the task, we recorded electromyographic signals from *extensor carpi radialis* and *flexor carpi radialis* muscles of the right arm, using a multichannel surface electromyography system (OTBiolab EMG-USB2+). For each muscle, two Ag/AgCl electrodes with an interelectrode distance of 26 mm were placed in parallel with the muscle fibers on the belly of the muscle. In an attempt to reduce crosstalk, standard electrode palcements were followed, as recommended [37]. The sampling frequency was 2048 Hz, with a gain of 1000, and an internal band pass filter with cut-off frequencies of [10–900] Hz. sEMG signals and kinematic data were synchronized through a trigger signal sent from the robot to the sEMG base unit to assure association between each muscle activation and the corresponding movement. The preparation for the test (namely, electrodes placement, grip force recording, adjustment of WRISTBOT height and oral instructions) required about 180 s; the duration of the test itself varied among subjects, mainly as a function of the total number of movements performed, with an average value of 80s (maximum duration in the overall population of subjects was 180 s). Globally, the assessment protocol could be performed with an average duration of 260 s and never exceeded 360 s.

### Data analysis

**Fig. 3:**
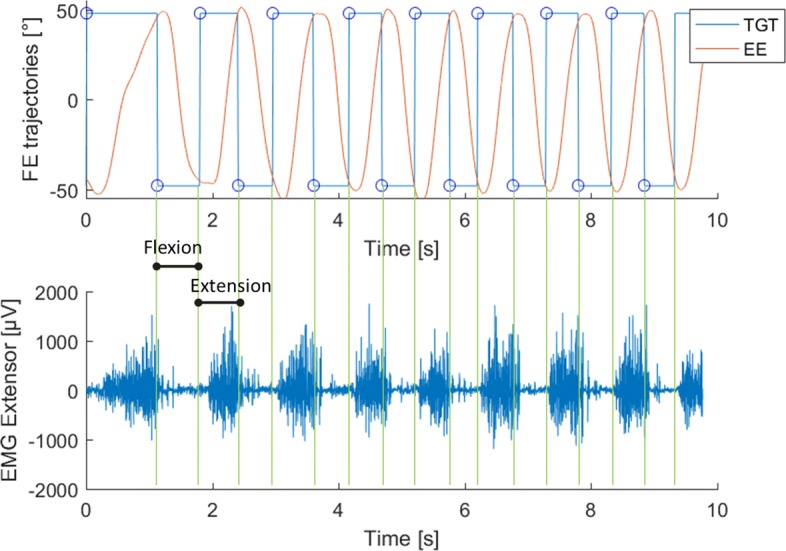
Example of data segmentation. **a**. Red lines represent an example of the end-effector trajectory in the flexion-extension plane to reach the target at ±48° (blue lines). **b** Example of sEMG signal of the *extensor carpi radialis* during the task. The signal was segmented according to the trajectory shown in Panel **a**

Wrist joint rotations, recorded from the robot encoders (data collection frequency set at 100 Hz), were converted to angular displacements and used to compute angular velocities. All kinematic data were processed with a sixth-order Savitzky-Golay low-pass filter (10 Hz cut-off frequency) and re-sampled at the sEMG sample rate (2048 Hz) by linear interpolation while sEMG data were band-pass filtered (5–350 Hz). sEMG and kinematic data were segmented to focus the analysis on the concentric phase for each group. The analysis of the trajectory data recorded by the robot allows for the extraction of each single flexion or extension movement as shown in Fig. 3 (Panel a and b). Accordingly, we analyzed the sEMG signal of *flexor carpi radialis* during flexion movements and the signal of the *extensor carpi radialis* during extension. Next, we computed a single value of the *Mean Frequency* for each of the obtained intervals of the sEMG signal. Therefore, N values of *Mean Frequency* (*F*_*Mean*_(*k*),*k*=1...*N*) were obtained for each subject with N being the total number of movements performed by the subject. In particular the *Mean Frequency* (*F*_*Mean*_) of a sEMG signal was calculated as follows:

2 $$ F_{Mean} =\frac{\int_{0}^{\frac{f_{s}}{2}}\! fP(f) \, \mathrm{d}f}{\int_{0}^{\frac{f_{s}}{2}}\! P(f) \, \mathrm{d}f}  $$

where *f*_*s*_ is the sampling frequency, and *P*(*f*) is the power spectral density (PSD) of the signal. In order to transform the sEMG signal from the time-domain to the frequency-domain, a Fourier transform of the autocorrelation function of the signal was employed and the PSD computed using the periodogram. The obtained N values of *Mean Frequency* of each subject, were fitted with a third order polynomial function based on mean least square approximations in order to calculate the *Onset of Fatigue (OF)*. This indicator was defined as follows: *OF* is the k-movement at which the initial *F*_*Mean*_ value of the sequence decreases by a given percentage (*P%*) (i.e. *F*_*Mean*_*(k=1)*). More precisely we used the following equation:

3 $$  {}OF_{P\%} \,=\, \!k\! \!:\! F_{Mean}(k) \!\leq\! F_{Mean}(1) - P\% \cdot\left(\!F_{Mean}(1)\,-\, min(\!F_{Mean}\!)\!\right)  $$

In order to choose the most appropriate value of *P%*, the acquired data were analyzed with three reference values, namely 25%, 50% and 75%, thus yielding *OF*_25*%*_,*OF*_50*%*_ and *OF*_75*%*_ respectively. Please note also that the decrement (*P%*), in Eq. 3, is calculated with respect to the minimum value in the *F*_*Mean*_ sequence which may not correspond, as will become evident in the “Results” section, with the final element of the sequence (*min*(*F*_*Mean*_)≠*F*_*Mean*_(*k*=*N*)).

To ascertain that muscular behavior was not due or related to changes in motor strategy we calculated for each movement two additional indicators based on movement kinematics: the *Time to velocity peak ratio* (*TPR*) and the *Mean speed* (m/s). The *TPR* is defined as the ratio between the time to velocity peak *TP*, i.e. the time from the beginning of the movement and the main peak of the speed profile, and the total duration of the main movement (*T*):

4 $$ TPR = \frac{TP}{T}  $$

Correlation analysis was performed to investigate the relationship between *Mean Frequency* and *Mean Speed* by evaluating the correlation coefficient (*CI*) between the two metrics. To constantly monitor the required effort throughout the task we evaluated the mechanical energy consumption in Joules for each subject for each movement, consisting of *N* samples, from the torque exerted (*τ*_*k*_) and the angular position (*θ*_*k*_) recorded by the robot, according to the following equation:

5 $$ E = \sum\limits_{k=1}^{N} \tau_{k} \cdot (\theta_{k+1} - \theta_{k})  $$

Such energy expenditure was converted into calories as to compare it with the basal metabolic rate. A statistical analysis was performed to investigate the possible significance of differences on the *OF*. *OF* data for the 40 subjects were z-transformed before the analysis. A post-hoc two-way ANOVA was chosen to investigate any difference in the *OF* between the three percentage levels in the two groups. The group factor (*FG*/*EG*) was set as “between” while, the *OF* percentage level (25/50/75) as “within”. Significant main and interaction effects were evaluated using a two tailed t-test with Bonferroni correction for multiple comparisons. Significance was set at p <0.05.

## Results

**Fig. 4:**
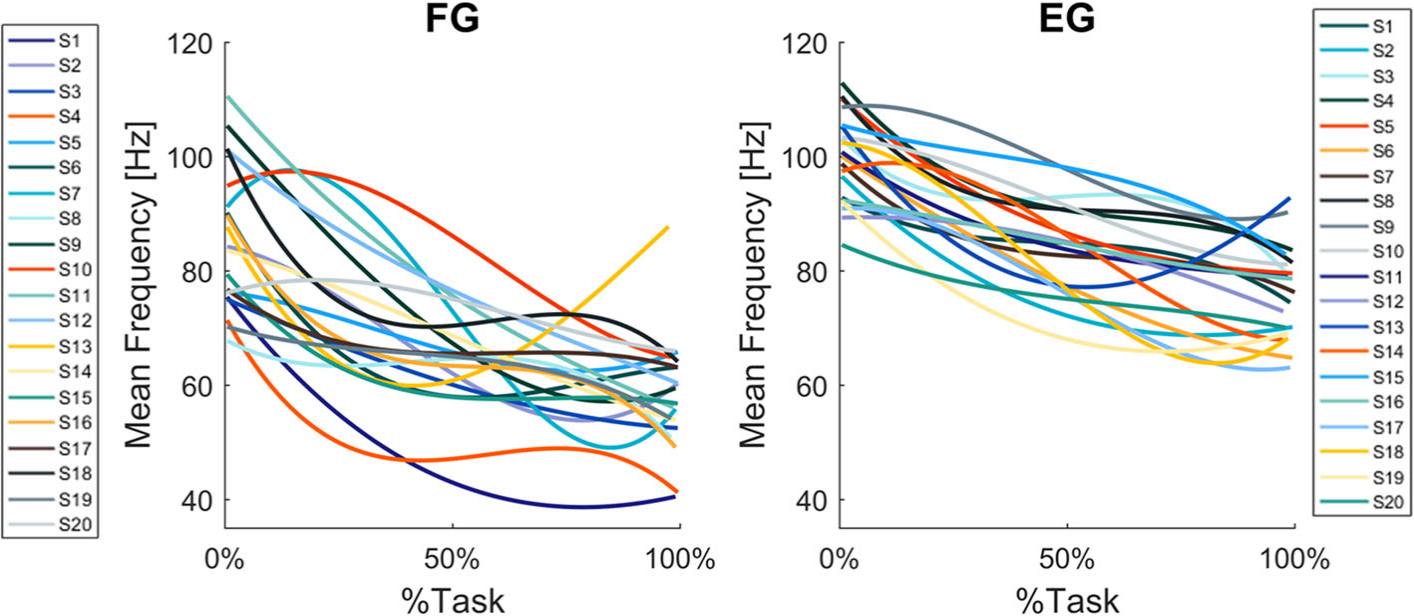
*Mean Frequency* results. Normalized *F*_*Mean*_ (Hz), of the *FG* (Panel **a**) and *EG* (Panel **b**), fitted with a third order polynomial function. Each line and color indicates a different subject

As expected, the *Mean Frequency* of both the *Flexion Group(FG)* and the *Extension Group(EG)* decreased during the first half of the task execution, and eventually reached a plateau (Fig. 4, Panel a and Panel b respectively), although in a few cases the decrease was not monotonic: consider, in particular, subjects S2-S4-S5-S6-S13 of the *FG* and subjects S13-S18-S19 of the *EG*. The fitting analysis indicated that the third-order polynomial model is a good predictor of the *Mean Frequency* trend, especially for the *Extension Group*. Indeed, 12 subjects out of 20 from the *EG* presented a goodness of fit higher than 0.6; such a goodness of fit was reached for 9 out of 20 from the *FG* (See Table 2).

**Table 2:** Goodness of fit (R^2^) and RMSE of *Mean Frequency* curves for each subject

	*FG*		*EG*	
	R^2^	RMSE	R^2^	RMSE
S1	0.89	3.7	0.33	5.37
S2	0.75	5.39	0.66	5.75
S3	0.74	3.94	0.20	8.06
S4	0.34	8.16	0.53	6.65
S5	0.21	8.41	0.76	4.94
S6	0.86	3.25	0.89	3.62
S7	0.73	11	0.66	3.57
S8	0.41	3.97	0.65	4.44
S9	0.91	4.53	0.83	3.32
S10	0.45	11.87	0.49	7.33
S11	0.71	9.78	0.58	5.04
S12	0.84	4.89	0.14	9.44
S13	0.49	8.40	0.79	3.73
S14	0.71	5.63	0.78	5.67
S15	0.37	7.36	0.23	9.78
S16	0.42	9.62	0.50	4.17
S17	0.35	3.94	0.61	8
S18	0.42	8.86	0.82	6.20
S19	0.47	3.94	0.67	5.08
S20	0.22	7.24	0.67	2.56

Goodness higher than 0.6 is underlined

**Fig. 5:**
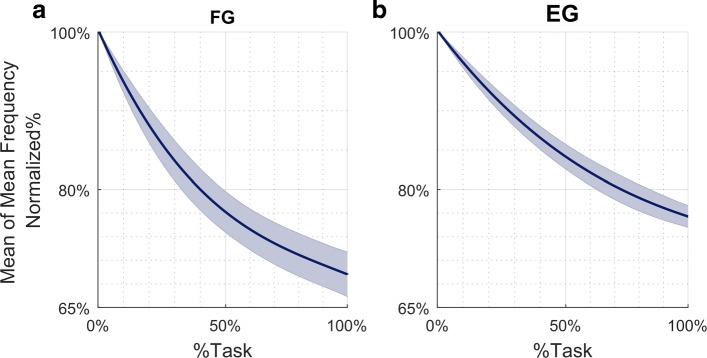
Averaged *Mean Frequency* results. Normalized *F*_*Mean*_ (Hz), of the *FG* (Panel **a**) and *EG* (Panel **b**), fitted with a third order polynomial function. Frequency was normalized to the initial frequency of each sequence and averaged across subjects. Shaded area represents standard error

Additionally, comparing the two groups, we found that the reduction in *Mean Frequency* was greater for the *flexor carpi radialis*, fatigued in the *FG*, than for the *extensor carpi radialis*, fatigued in the *EG*. Specifically, Fig. 5 represents the evolution of the averaged *Mean Frequency* across *FG* and *EG* subjects (panel a and b respectively), normalized with respect to the initial value. From such a curve, it is possible to evaluate the percentual decrease of the averaged *Mean Frequency* of the fatigued muscles, with respect to the beginning of the task execution: 31% for the *flexor* and 24% for the *extensor*.

**Fig. 6:**
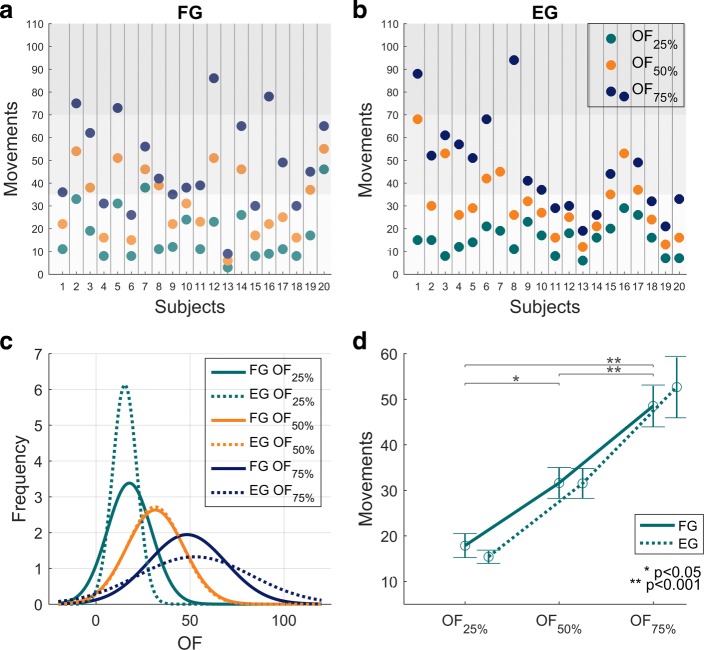
*OF* results. Results related to *OF* indicators (Onset of Fatigue). Panels **a** and **b** show, for each *FG* subject (panel **a**) and *EG* subject (panel **b**), the values of the three *OF* indicators: *OF*_25*%*_,*OF*_50*%*_,*OF*_75*%*_, expressed as number of “movements” that satisfy Eq. 3. Grey areas divide the task in three phases. Panel **c** shows the Gaussian approximation of the probability density functions of the three *OF* indicators for the *FG* population (solid lines) and *EG* population (dashed lines). Panel **d** shows the mean values and standard errors of the three *OF* indicators for the *FG* population (solid line) and the *EG* population (dashed line)

The next step of the analysis was to compare the three criteria for the identification of the *Onset of Fatigue* (namely, *OF*_25*%*_,*OF*_50*%*_ and *OF*_75*%*_) in order to identify the most reliable approach, in view of application in daily clinical practice. The experimental results indicate that *OF*_25*%*_ occurred almost at the same time in both groups, suggesting higher consistency of this parameter, among subjects, than *OF*_50*%*_ and *OF*_75*%*_. *OF*_25*%*_ occurred within the first 25 movements (see Fig. 6 Panels a, b, c) independently of the number of movements performed by each subject (see Table 3); *OF*_50*%*_ occurred around movement number 30 and *OF*_75*%*_ around movement number 50. The main difference among the three *OF*s was evident in reliability: Figure 6 Panel c displays the Gaussian approximation of the probability density functions of the three *OF* indicators for the *FG* population and *EG* population, respectively: *OF*_25*%*_ provides a more reliable estimate of onset of fatigue because it is characterized by a much smaller variance than the other two, with *OF*_75*%*_ being the least robust and most variable. It is worth mentioning that, despite a higher variance than *OF*_25*%*_,*OF*_50*%*_ presented the highest consistency between the two groups, as indicated by the two orange Gaussian functions (Fig. 6 Panel c) that are almost identical. These results are confirmed by the statistical analysis which revealed significant differences among the three *OF*s (F(2, 114)=35.485, p <0.001) independently from the group. There was no interaction between *OF* and the group (F(2, 114)=0.34286, p=0.71046). There were no significant differences between the two groups for the number of movements to *OF* (shown in Fig. 6 Panel d) (F(1, 114)=0.02614, p=0.87183). However, post-hoc analysis demonstrated a significant difference between *OF*_25*%*_ and *OF*_50*%*_ (p=0.001), between *OF*_25*%*_ and *OF*_75*%*_ (p <0.001), and between *OF*_50*%*_ and *OF*_75*%*_ (p <0.001).

**Table 3:** *Mean speed - Mean Frequency* correlation coefficients

	*FG*	*EG*
S1	-0.68	-0.23
S2	-0.68	-0.54
S3	-0.37	0.04
S4	-0.39	-0.22
S5	-0.49	-0.61
S6	-0.54	0.01
S7	-0.58	-0.56
S8	-0.51	0.09
S9	-0.58	-0.56
S10	-0.01	-0.02
S11	-0.74	0.24
S12	-0.79	-0.06
S13	-0.06	-0.23
S14	-0.44	-0.22
S15	-0.26	-0.17
S16	-0.46	0.07
S17	-0.19	-0.28
S18	-0.17	-0.37
S19	0.15	-0.61
S20	0.34	-0.42

**Fig. 7:**
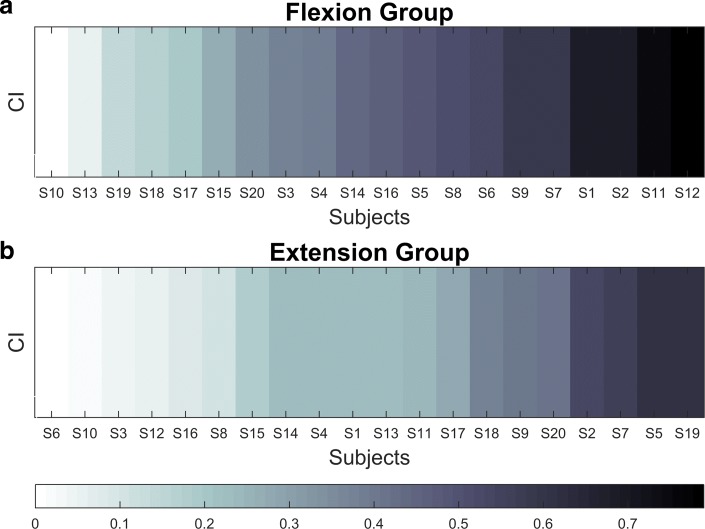
CI results. Absolute values of the *Mean speed* and the *Mean Frequency* correlation for the *FG* (Panel **a**) and the *EG* (Panel **b**. Subjects are sorted by correlation, as indicated in the bottom panel legend

The kinematic analysis of the performed experiments should demonstrate any potential relationship between a subject’s motor strategy and patterns of muscle activity. In particular, we evaluated the correlation coefficient between the *Mean speed* and the *Mean Frequency* (*CI*), and this is reported in Table 4 and Fig. 7 (absolute values). Overall, the correlation is negative, namely higher *Mean speed* implies lower *Mean Frequency*, but, in general, it was a weak relationship: on average, the absolute correlation value was 0.42 for the *FG* population and 0.27 for the *EG* population, and it never exceeded 0.8.

**Table 4:** Number of movements performed by each subject and total calorie consumption

	*FG*		*EG*	
	N° Mov	Cal	N° Mov	Cal
S1	94	70	97	55
S2	145	109	133	113
S3	104	62	66	50
S4	104	81	117	102
S5	146	103	105	64
S6	101	59	110	82
S7	86	73	209	137
S8	44	40	117	80
S9	73	60	66	37
S10	48	51	54	43
S11	61	40	60	22
S12	126	117	34	22
S13	38	18	67	39
S14	89	74	34	28
S15	105	90	49	37
S16	92	70	114	83
S17	150	106	75	51
S18	104	92	56	49
S19	49	31	57	33
S20	78	51	48	28

**Fig. 8:**
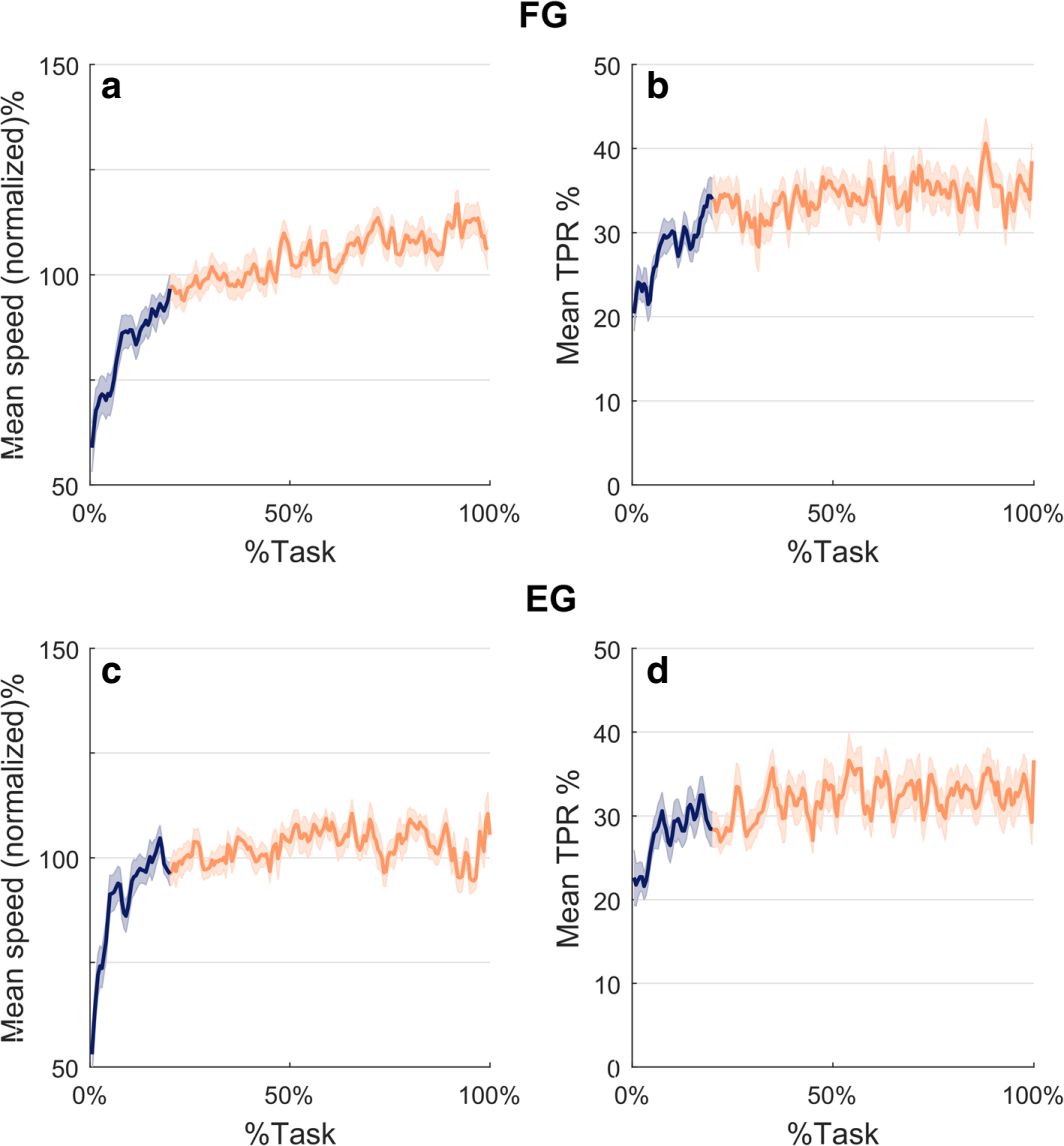
Kinematics results. Panel **a** and **c**: *Mean speed* normalized by the mean of each subject and averaged over *FG* and *EG* subjects respectively. Panel **b** and **d**: *Time to peak ratio* averaged over *FG* and *EG* subjects respectively. %Task identifies the relative ordinal number of the task-movements of all the sequence. In all panels shaded areas indicate the standard error, the blue portions refer to the first 20% of the task while the red corresponds to the remaining 80%

In spite of the low correlation between speed and frequency, the kinematic analysis was valuable for demonstrating the consistency of the designed protocol. This is shown in Fig. 8 which plots the evolution during task execution of the normalized *Mean speed* (panels a and c) and the *Time to velocity peak ratio* (panels b and d). The *Mean speed* graph is characterized, for both groups of subjects, by a steady increase in the first 20% of the task execution (blue portion of the curve) followed by a plateau in the remaining 80% of the task. The graph of the *Time to velocity peak ratio* (*TPR*) is characterized by a similar trend, with an initial transient related to the first 20% of the task execution, followed by a plateau for the rest of the task. This suggests that there were no changes in wrist kinematic strategies. It is worth noting, there was a difference between the two groups. The *FG* subjects exhibited a speed profile with higher symmetry as indicated by the fact that *TPR* is closer to 50% than for the *EG* subjects.

Finally, the analysis of the energy consumption revealed that the average number of calories consumed during the task was 63.80 ± 4.75 calories (mean and sem). Individual values are shown in Table 4.

## Discussion

The importance of muscle fatigue assessment in patients with neuromuscular disorders has long been recognized. Specifically, a precise measure of muscle fatigability could provide crucial information in diagnosis, treatment planning and evaluation of therapy efficacy. Yet, there is a lack of quantitative standardized and reliable methods for fatigue assessment used in clinical practice and these may not be feasible for all pathological populations at various stages of a disease. Muscle fatigue assessment using surface electromyography signals has been widely studied under isometric conditions [38] and in particular during maximum voluntary contractions [18, 20, 21]. However, the capacity to voluntarily generate and maintain a maximal force in isometric conditions might be limited by a lack of motivation and it could represent a highly demanding task for people affected by severe weakness or injury. When dealing with adolescent populations, it is common to see a lack of full cooperation, which makes it challenging to measure (in a reliable manner) maximum contraction force and consequently muscle fatigue [25]. Furthermore, it is worth highlighting that subjects with low residual motor functions are hardly ever able to perform MVCs. Hence, few tests based on dynamic exercises have been proposed and validated [39, 40], thus representing a consistent alternative to isometric contractions. Based on these considerations, our approach aimed to evaluate muscle fatigue, regardless of individual motor capabilities. In particular, the use of a robotic task allows for the experimenter to tailor the evaluation, in term of range of motion and force required, to an individual subjects capability and strength, thus allowing for adoption by a large patient population. Additionally, the adoption of a method relying on robot mediated movements assures a more controlled and repeatable execution of the test than currently used isometric or dynamic exercises. A preliminary, but different, version of such a test has already been validated in a pilot study which revealed its feasibility and repeatability [31]. As an extension and consolidation of that preliminary study, in this paper we report the results obtained with 40 healthy subjects tested with a novel improved version of the robot-based assessment protocol. In particular, we used a visco-elastic force instead of a pure viscous force field since, with neuromuscular patients, a force that depends on velocity could reduce the repeatability of the results. In addition, we reduced the effort required to perform the test, with a resistive visco-elastic force in one direction. This work revealed that the proposed test is easy and fast to administer, provides an objective and reliable measure of muscle fatigue and can be used in a clinical setting. It has also to be mentioned that the use of the robotic device adds the ability to measure subjects’ performances in terms of kinematic parameters, thus resulting in a more detailed assessment of the patient. In the present study, the kinematic analysis demonstrated the stability of the *OF* indicators and it appears robust, given different motor control strategies. Regarding the applicability of the method, the experimental setup is minimal, requiring sEMG from two target muscles and the correct alignment between the human wrist and the robotic device. From a clinical perspective, test duration is also important and our test never lasted more than 3 min. It is even reasonable to expect a shorter test time in clinical populations compared to our healthy participants. We chose to base our indicator of *Onset of Fatigue* on the *Mean frequency* since its variance is tipically lower than that of *Median frequency* [41]. The shift in *Mean frequency* towards the lower frequency spectrum was noticeable in both the *flexion* and *extension* groups, however the shift was greater in the former. This may be due to different physiological properties of the two muscles: I) from a biomechanical perspective of the human wrist joint, the amplitude of the range of motion in flexion is higher than that achievable in extension (peak flexion moment is approximately 70% higher than peak extension moment [42]); II) the percent decrement of *Mean Frequency* is proportional to the amount of catabolites produced by muscle fibers during activity [43]. In particular, the quantity of catabolites depends on the average number of muscle fibers per square unit of the muscle crossection [43] and consequently the higher the crossection, the higher the amount of catabolites and the greatest the rate of decrease of the *Mean Frequency* ; III) muscle fiber type will influence sEMG parameters, in particular, a greater percentage of type II fibers leads to a greater rate of decrease of the *Mean Frequency* [44]. As reported from other studies, muscles belonging to the extensor group fatigue more and faster than flexors [45], therefore, *EG* population was expected to exhibit more fatigue. However, directly comparing the single muscles *extensor* and *flexor carpi radialis*, the physiological cross-sectional area (PCSA) of *flexor carpi radialis* is about half the size of the *extensor carpi radialis* [46]. Therefore, we can assume that *flexor carpi radialis* has a lower force generating capacity. We can speculate that, on a whole, when using the same force field intensity for both flexion and extension, the extensor muscles would fatigue more than the flexors. It should be noted that we measured only one wrist extensor muscle. The most important contribution of this method is the development of and evaluation of the *OF* measure. The *OF* in the *EG* presented less variability compared to the *FG*, probably because of the different muscle properties mentioned above. As for the optimal version for *Onset of Fatigue*, the comparison among *OF*_25*%*_,*OF*_50*%*_ and *OF*_75*%*_ revealed that *OF*_25*%*_ is the more consistent and less variable. We can postulate that after an initial decrease, which is very similar in both groups, the *Mean Frequency* curves decreased with different slopes due to different subject training levels and muscle physiological properties. Our *OF*_25*%*_ is also more inline with previous studies that have suggested that a mean frequency decrease of 8% is representative of muscle fatigue onset [47, 48]. Moreover, our subjects’ ability to perform the task correctly from the very beginning and the consequent stability of the kinematic parameters, support the adoption of *OF*_25*%*_ as an indicator of fatigue. *Mean speed* stabilized in the first 20% of the task, suggesting that *Mean Frequency* and *OF*_25*%*_ are not related to kinematic changes. It is worth noting that, in some cases, during the last part of the task, the trend was inverted. From a physiological perspective, this may be related to a de-recruitment of fatigued motor units, in favor of the recruitment of new motor units [24]. This finding is also in line with previous studies showing that, during submaximal contractions, motor units recruitment can still increase when motor units start to be fatigued, while during maximal contractions such a rise is limited [49, 50]. On the other hand, we are aware that the frequency recovery could be due to the effect of cross-talk between adjacent muscles. However, since we collected from the *flexor* and the *extensor carpi radialis*, further investigations recording additional muscles are needed to examine the potential effect of cross-talk during our task. Additionally, it has been reported that an increase in muscle temperature leads to an increase in *Mean Frequency* [51]. Therefore, future work might consider measuring surface temperature or muscle temperature to investigate any potential relationship between an increase in temperature and the inversion of the *Mean Frequency* trend observed in our study. Regarding kinematic measurements, we found that the *Mean Speed* stabilized and remained constant after an initial phase, corresponding to the first 20% of the task, in which it increased up to a plateau level. This suggests that *Mean Speed* was not affected by muscle fatigue (and vice-versa), which is in line with previous finding [52–54]. Conversely from what was expected, we did not find changes in kinematic strategies that correlated with increasing fatigue level. Specifically, the *TPR* did not show a shift in the peak of the bell-shaped speed profile [55] from early trials to the late trials in which fatigue appeared. A final aspect to consider was task duration. In the proposed protocol, the number of repetitions performed, was decided by the subjects and not superimposed by the experimenter. Thus, subjects were instructed to stop when they felt tired, which is crucial in a clinical scenario. The number of repetitions, therefore, could also be considered as an additional measure of performance [56], especially for populations with neuromuscular impairments, where kinematics and sEMG might have to be cautiously interpreted. Our results in healthy participants demonstrate that *OF* was independent from the amount of repetitions of the reaching movements performed. This may be a consequence of the population studied, who could tolerate a high level of resistance and may not stop the test when they feel fatigue. To conclude, the developed algorithm could be improved in the future by measuring individual wrist strength and grip force throughout the task [45]. Our approach used two levels of force, according to sex, but customizing the force and normalizing to individual maximal force production, could improve *OF* results. This aspect is particularly relevant and needs to be considered in the application of this method to pathological subjects. Lastly, this study suggested that a fatigue assessment coupling a robotic task and sEMG recordings is highly feasible and practical. The present study provided a good starting point for the application of the test in clinical practice, however pilot experiments with neuromuscular and age-matched healthy subjects are necessary to confirm the results. Finally, wrist robotic device guarantees the repeatability of the task, providing the same force and trajectory. Moreover without the addition of further measurement tools we attempted to exploit the torque and angular position data recorded by the robot as a simple measure of the mechanical work performed by the subject during the test. Although such an approach does not provide specific information about actual internal muscle work or physiological work, it allowed for an estimate of the total energy required by the task. Our test has been demonstrated to require little effort, so the impact on daily energy expenditure (avg 2500–3000 kcal) would be minimal [57].

## Conclusion

This test will provide clinicians with an objective and easily readable indicator of muscle fatigue. The method is simple, easy to administer and suitable for patients/participants who are not able to generate high levels of muscle contractions. This overcomes the problem of administering maximal contraction tests in neurological or injured populations. If the same robust results could be obtained from pathological population, then this method could be used as a standard muscle test procedure that is independent of the subjects ability or willingness to perform voluntary efforts. In this context, the final perspective of the use a robotic device is to assess muscle fatigue in very controlled conditions and with the possibility to change or adapt the task to the population needs.

## Abbreviations

CI: Correlation index
DoF: Degree of freedom
EG: Extension group
FE: Flexion/extension
FG: Flexion group
OF: Onset of fatigue
PS: Pronation/supination
RoM: Range of motion
RUD: radial/ulnar deviation
TPR: Time to velocity peak ratio
